# Research Progress of Cholesteric Liquid Crystals with Broadband Reflection

**DOI:** 10.3390/molecules27144427

**Published:** 2022-07-11

**Authors:** Huimin Zhou, Hao Wang, Wanli He, Zhou Yang, Hui Cao, Dong Wang, Yuzhan Li

**Affiliations:** School of Materials Science and Engineering, University of Science and Technology Beijing, Beijing 100083, China; zhm10051766@163.com (H.Z.); ther8437@163.com (H.W.); caohui@mater.ustb.edu.cn (H.C.); dwang.lgh@gmail.com (D.W.); yuzhanli@ustb.edu.cn (Y.L.)

**Keywords:** liquid crystals, cholesteric phase, selective reflection, broadband reflection, pitch gradient, non-uniform pitch distribution

## Abstract

Cholesteric liquid crystal (ChLC) materials with broadband reflection are witnessing a significant surge in interest due to their unique ability to self-organize into a helical supra-molecular architecture and their excellent selective reflection of light based on the Bragg relationship. Nowadays, by the virtue of building self-organized nanostructures with pitch gradient or non-uniform pitch distribution, extensive work has already been performed to obtain ChLC films with a broad reflection band. This critical review systematically summarizes the optical background of the ChLCs with broadband reflection characteristics, methods to obtain broadband reflection of ChLCs, as well as the application in this area. Combined with the research status and the advantages in the field, the challenges and opportunities of applied scientific problems in the research direction are also introduced.

## 1. Introduction

Cholesteric liquid crystals (ChLC) is of great interest to researchers due to their special self-assembled helical structure and selective light reflection properties. The cholesteric phase is considered as a special state of the nematic phase and is also often referred to as chiral nematic liquid crystals [1]. Unlike the mainly oriented sequential character of the nematic liquid crystal phase as an elongated molecule, ChLCs exhibit a spontaneous helical structure with the twist axis perpendicular to the local orientation [2,3,4]. The ChLC molecules are arranged in layers, with the long axis of the molecules parallel to the layer planes and each plane rotated at a certain angle with respect to its neighboring planes [5]. The pointing vector of liquid crystal molecules in the layer direction returns to the initial orientation state after 360° of rotation, and this periodic layer spacing is called the pitch (P) [6,7]. The pitch can change with the chemical environment, temperature, electric field, and so on.

Due to the unique helical structure of ChLCs, they exhibit special optical properties, such as selective light reflection [8,9], circular dichroism [10,11], and rotational properties [12,13], which make them widely used in various fields. In recent years, the selective reflection of ChLCs has become a hot research topic of wide interest. ChLCs with reflection wavelength in the visible region can be applied to temperature indication [14], liquid crystal display panel light brightening film [15], anti-counterfeit trademark [16], mirrorless low-threshold laser [17,18], etc. ChLCs with reflection wavelengths in the near-infrared region can be applied to energy-saving and environment-friendly architectural glass or paint [19], etc. ChLCs with reflection wavelengths in the mid-infrared and far-infrared regions have potential applications in military shielding and stealth [20]. Thus, ChLCs with broadband reflective properties have significant appeal and wide market demands due to their outstanding characteristics. However, single-pitch ChLCs tend to have a reflection width less than 200 nm due to their excellent selective reflection of light based on the Bragg relationship [21]. How to realize the broadening of the reflection bandwidth within the desired reflection spectrum is still one of the critical problems to be solved for the application of ChLCs. In this paper, we summarize the progress of research on ChLC materials with broadband reflection in recent years. The in-depth study of the broadening method and mechanism will help to further develop new optical liquid crystal materials and expand the applications of ChLCs. 

## 2. Broadening the Reflection Bandwidth

Because of the periodic variation in refractive index caused by the helical structure, ChLCs can selectively reflect the incident light along the helical axis direction. Due to the circular dichroism of the ChLC structure, its selective reflection is not independent of the linearly polarized light, but only the circularly polarized light with the same rotation direction as the ChLC structure. Therefore, the left-handed or right-handed ChLC reflection incident light is limited to 50%. ChLCs with a single-pitch selectively reflect the light of a wavelength between *λ_min_* = *Pn_o_* and *λ_max_* = *Pn_e_*. Here, *n_o_* and *n_e_* are the ordinary and extraordinary refractive indices of the locally uniaxial structure, respectively. The bandwidth of the selective reflection spectrum ∆*λ* is given by ∆*λ* = *λ_max_* − *λ_min_* = (*n_e_* − *n_o_*) *P* = ∆*nP*. Here, ∆*n* = *n_e_* − *n_o_* is the birefringence. According to the above equation, we can see that the bandwidth ∆*λ* is dependent on the birefringence ∆*n* and the pitch *P* at normal incidence. Since the birefringence Δ*n* of ChLC materials is generally less than 0.4 [20], the bandwidth of a ChLC with a single-pitch selective reflection of incident light is narrow (generally less than 200 nm), and it is difficult to adjust the bandwidth by adjusting Δ*n* [22,23]. Therefore, it is necessary to adjust the pitch gradient or the non-uniform pitch distribution in the ChLC material system to achieve the broadening of the reflection bandwidth. 

As we all know, ChLCs are usually observed in liquid crystal molecules containing chiral units or obtained by adding chiral compounds to nematic liquid crystals. However, the pitch of small molecule ChLCs is usually uniform, single, and narrow due to their low viscosity. Therefore, in order to obtain the broadband reflection of a ChLC, it is usually achieved in a polymer/ChLC system. This may be due to the high viscosity of polymer and the fixed network structure, which limit the diffusion between different length pitch structures, so as to form microstructures with different length pitch distributions. According to the monomer content, the polymer/ChLC system can be divided into small-molecule ChLCs (without monomer), cholesteric liquid crystalline polymers (with 100% monomer), and polymer-stabilized ChLCs (with a small amount of monomer). ChLC polymers are completely polymerized by liquid crystal monomers containing chiral units. Regardless of the mesogenic units in the main chain and side chain, ChLC polymers have high molecular weight and high mechanical properties. Due to the moderate to highly crosslinked network, the ChLC polymer network retains only a small amount of ChLC properties, replicating the helical structure of ChLC in space [24]. Due to high viscosity or the fixed network structure, the orientation and diffusion of ChLC polymers are almost unchanged when stimulated [25]. Polymer-stabilized cholesteric liquid crystals (PSCLC) have a large number of small-molecule ChLCs as continuous phases and the mass fraction of the polymer is usually less than 10%, which can be formed by the polymerization of mesogenic monomers or the monomers without mesophase [26]. Due to the low content of polymer monomers, PSCLC can not only form polymer networks, but also properly retain the ChLC characteristics, which plays a key role in the formation and fixation of microstructure with pitch gradient distribution or uneven pitch distribution. Herein, we will introduce the approach to broaden the reflection bandwidth from the following aspects: multilayer system, responsive chiral molecules, light-controllable polymerization rate, thermally induced molecular diffusion, two-phase coexistence material system, memory effects of the template, and electromagnetic-induced molecular diffusion.

### 2.1. Multilayer System

**Figure 1:**
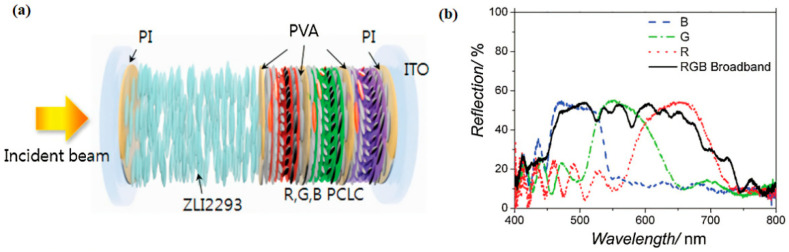
(**a**) Structure of a device consisting of multilayered PCLC film and a NLC layer. (**b**) A broadband refection spectrum (black solid curve) consisting of each reflection spectrum of R, G, and B PCLC films [28]. Reproduced with permission from Choi H, Adv. Mater.; published by John Wiley and Sons, 2010.

This method of multilayer system is mainly used to broaden the reflectance bandwidth of the composite system by superimposing ChLC samples with different pitches. Firstly, multiple polymer/liquid crystal films with different pitches should be prepared. Usually, copolymerization of different amounts of chiral monomers or doping of different chiral compounds into PSCLC are used to form multiple ChLC polymers or PSCLC films that can reflect incident light of different wavelengths, respectively. Then, ChLC film layers with different pitches are stacked together in a certain order. Finally, the stacked films are bonded or polymerized together to obtain a reflection band wider than any film layer. Usually, the obtained reflection band is between the maximum and minimum reflection wavelengths in each ChLC film. Kralik obtained a ChLC film reflected wavelength that covers the visible light region, by superimposing three layers of ChLCs reflecting red light, green light, and blue light, respectively [27]. Choi manufactured a continuous wide reflective band by superimposing three right-handed polymer cholesteric liquid crystal (PCLC) layers with different pitches and a sandwich structure of nematic liquid crystals (NLCs) made up of these PCLC films. Samples of PCLC with different reflection bands were spin-coated on the substrate, in turn, with the PVA film acting as an alignment layer for the upper PCLC layer and a barrier layer preventing the lower PCLC layer from being dissolved by the new PCLC film stack. Through this stacking process, broadband multilayer stacked PCLC films were obtained. A broadband reflective film covering the visible light range was obtained by stacking RGB PCLC films (as shown in Figure 1). While each PCLC layer provides a smooth reflection band, the multi-layer stacked layers exhibit some degradation in the reflection band. This phenomenon is due to defects in abrupt pitch shifts in PCLC layers and inserted isotropic polymer layers [28]. 

**Figure 2:**
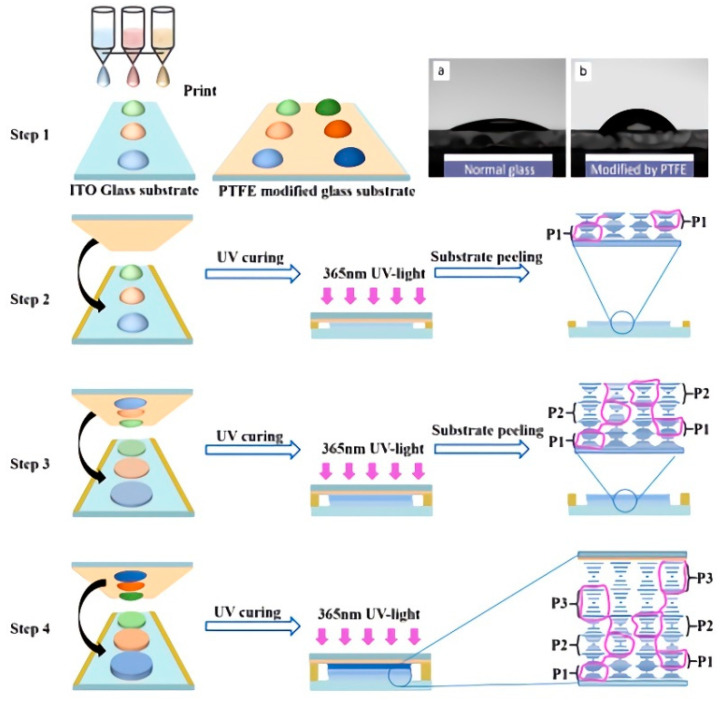
The scheme of a broadband reflective film prepared by inkjet printing.

Recently, a new approach to the preparation of broadband reflective ChLC films based on inkjet printing and non-stick technology was proposed in our research (as shown in Figure 2) [29]. We filled the separate C, M, Y channels with chiral molecules, polymerizable monomers and liquid crystals, respectively, and controlled the inkjet volume by software (Acro-rip). In order to prepare a broadband reflective film by stacking layers, we separately printed the precursor liquid crystal layer on each substrate with different chiral additions. After UV polymerization of the first liquid crystal layer, the second liquid crystal layer was transferred to the first liquid crystal layer for polymerization. Then, a third, fourth, or even more layers were stacked in the same way and the thickness of each PSCLC film was controlled by adding polyimide films. The advantages of the single-pitch multilayer stacking method are that the preparation process is very simple and the wavelength and the reflection range of each film are controllable. However, the stacked multilayer system is not a direct combination of monolayer characteristics, and there is certain diffusion between the film interfaces, which, to a certain extent, affects the plane orientation of ChLC molecules in the interface, resulting in low reflectivity and transmittance of the final obtained films. In addition, the thickness of the film obtained by the above method is relatively thick, and does not have the function of dynamic adjustment, which reduces the practicability.

### 2.2. Responsive Chiral Molecules

ChLCs with helical structures can be formed by adding chiral molecules to the nematic phase liquid crystals. Helical twisting power (HTP) represents the ability of chiral molecules to induce the formation of helically arranged structures in nematic liquid crystals, which is directly related to the pitch of the helical superstructure of ChLCs (HTP = (PX_c_)^−1^). Here, P and X*_c_* are pitch and chiral molecule concentration, respectively. Therefore, the HTP of the chiral molecule plays a decisive role in the pitch of ChLCs. The responsive chiral molecule allows a change in HTP of the chiral molecule under different external fields (thermal, optical), which changes the pitch of the ChLCs and allows for the adjustment of the reflection wavelength [30]. 

Duan synthesized a responsive chiral compound of which the HTP decreases with increasing temperature and formed a PSCLC film with a pitch gradient distribution by using two polymerizable monomers [31]. One is 1,4-di- [4-(6-acryloyloxy) hexyloxy benzoyloxy]-2-methyl benzene (C6M), a free-radical polymerizable monomer with high polymerization activity at low temperatures, and the other is ethylene glycol diglycidyl ether (EDGE), a cationic polymerizable monomer with high polymerization activity at high temperatures. Based on the large HTP temperature dependence of a chiral dopant, polymer networks with different pitches were formed by polymerizing C6M to fix a small pitch at low temperature and EDGE to fix a large pitch at high temperature in sequence. The research by Zhang confirmed that an appropriate concentration of free-radical monomers and a sufficient concentration of cationic monomers are essential for the formation of PSCLC films with broadband reflectivity [32]. Furthermore, it was shown that the increased functionality and rigidity of the cationic monomers had a positive effect on the broadening of the reflection bandwidth. 

**Figure 3:**
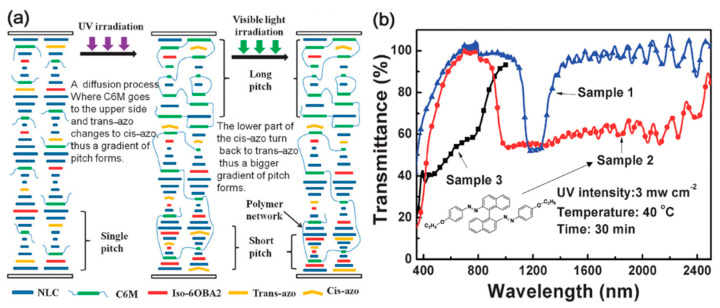
(**a**) The manufacturing process and possible mechanism of the broadband reflection of a PSCLC doped with the chiral azo compound. (**b**) Transmittance spectra of material systems with (sample 2) or without (sample 1) the chiral azo compound [33]. Reproduced with permission from Chen X W, Chem. Commun.; published by Royal Society of Chemistry, 2014.

Due to the rapid development of photoresponsive chiral compound materials, the modification of the HTP of chiral compounds by light, combined with the PSCLC to fix the distribution of the pitch gradient, has become a new approach to broaden the reflection bandwidth of ChLC films. As shown in Figure 3, Yang reported the azobenzene chiral compound could induce pitch gradient distribution by combining UV absorption properties and altering the HTP of chiral azobenzene compounds for the first time [33]. When light is irradiated from top to bottom, a gradient distribution of light intensity is generated in the direction of liquid crystal cell thickness. The degree of molecular isomerization along the thickness direction is distributed in a gradient, leading to the distribution of the pitch gradient. The presence of the polymer network causes the fixation of the helical structure. The network density formed from top to bottom gradually decreases, leading to a significant difference in the effect of azo–chiral compound replies and further realizing the broadening of the reflection bandwidth. The reflection wavelength of the composite can be expanded to 1000–2400 nm. Lu synthesized a new cyclic chiral azobenzene compound, Azo-o-Bi, which shows high HTP and optochemically reversible cis-trans isomerization in organic solvents and liquid crystals. Due to the strong absorption of UV light by the chiral Azo-o-Bi molecule, the cis-isomers with high HTP accumulated on the side close to the UV irradiation. The gradient distribution of the pitch in the direction of UV propagation induced a broadband reflection ChLC film [34]. 

**Figure 4:**
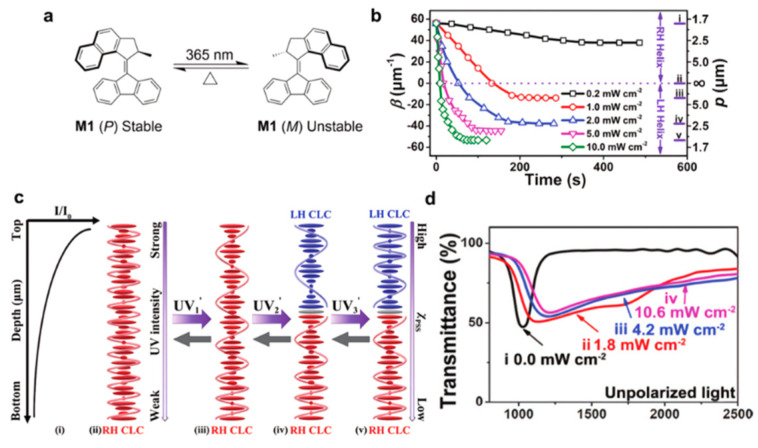
(**a**) Molecular isomerization of overcrowded alkene M1 under UV exposure. (**b**) Variation in the HTP value (β) and pitch length (p) of a M1 (1.0 wt%)/LC513 mixture upon exposure to different UV intensities. (**c**) Schematic illustrations of the photodynamic ChLC molecular arrangements in ChLC cell with different UV-irradiation intensity (UV_1_ < UV_2_ < UV_3_). Here, (i) UV intensity distribution across a thicker film, where I0 is the original intensity of UV light and I is the intensity at the corresponding film depth; (ii) the uniform helical pitch of the ChLC in the initial state before irradiation; (iii) the gradient distribution of ChLC pitches in the same right-handed (RH) direction; (iv) the pitch gradient distribution of the left-handed (LH) and RH ChLC coexistence; (v) the symmetrical pitch gradient distribution of the LH and RH ChLC coexistence. (**d**) The transmittance spectra of the ChLC mixture driven by different light intensities [35]. Reproduced with permission from Sun J, J. Mater. Chem. C.; published by Royal Society of Chemistry, 2017.

Yang constructed broadband reflectivity and super-reflectivity by exploiting the strong UV-intensity dependence of the photo-isomerization of chiral molecular motors (as shown in Figure 4) [35]. Under different light intensities of UV irradiation, the HTP of chiral molecules can be significantly reduced, and the stronger the light intensity, the greater the change in HTP values. By irradiation with different light intensities of UV light, the pitch and handedness of the ChLCs change accordingly. The gradient distribution of the light intensity in the direction of the cell induces a pitch gradient in the ChLCs, resulting in a broadband reflection with a bandwidth of 1080–2050 nm. 

### 2.3. Light-Controllable Polymerization Rate

Polymerization rate is one of the important mechanisms to obtain pitch gradient or a non-uniform distribution of pitch in ChLC materials. As the structure of the molecular arrangement of the LC phase can be fixed by the photopolymerization reaction of liquid crystalline polymers, the polymerization rate of monomers can be adjusted by controlling the UV light intensity in the direction of liquid crystal cell thickness, thus, realizing the regulation of ChLC pitch. The stronger the light irradiation, the faster the monomer polymerization and the more monomer consumption. The non-chiral monomer diffuses in the direction of more consumption, which drives the chiral molecules to diffuse in the opposite direction to form a pitch gradient. However, when the rate of monomer polymerization is too fast, the monomer is no longer dominated by diffusion but preferentially fixed, which is not conducive to the formation of pitch gradient. In addition, other factors, such as polymerization monomer type, UV absorbing dye concentration, photoinitiator, sample thickness, polymerization temperature, UV light intensity, and other experimental conditions, have important effects on the polymerization rate, and adjusting these parameters can effectively regulate the reflection wavelength and bandwidth [36,37].

This method of light-controllable polymerization rate was first proposed by Dutch scientist Broer [38]. The material system of bifunctional chiral liquid crystal polymerizable monomer, monofunctional liquid crystal polymerizable monomer, ultraviolet light absorbing dye and photoinitiator was used for the first time. UV intensity gradient occurs in a certain direction due to the existence of the dye. The top of the cell polymerizes at a faster rate than the bottom. The polymerizable monomer diffuses to the top and chiral molecule diffuses to the bottom accordingly, thus, forming a pitch gradient. The reflectance spectrum can be effectively broadened by using multiple gradients of UV-induced polymerization in ChLCs and controlling the rate of photopolymerization. The intensity gradient of the UV light varies depending on the distance between the UV lamp and the cell, which affects the polymerization rate and leads to the formation of spiral structures with different pitches [39].

In addition, it was found that certain liquid crystal materials possess UV light absorption properties inherently. It is possible to create UV intensity gradients in the direction of the liquid crystal cell thickness without the use of dye, thus, triggering differences in polymerization rates. Mitov found that asymmetric irradiation of liquid crystal cassettes with weak UV light in small molecule liquid crystals, bifunctional liquid crystal polymerizable monomers, and photoinitiator material systems can induce pitch gradients in polymer networks. The concentration of the polymer network is higher near the UV source and lower away from it, resulting in a significant broadening of the selective reflectance from 80 nm to 220 nm, while the polymer network produced under symmetric irradiation has no significant gradient distribution and the reflection bandwidth is narrower than that of asymmetric irradiation [40]. 

**Figure 5:**
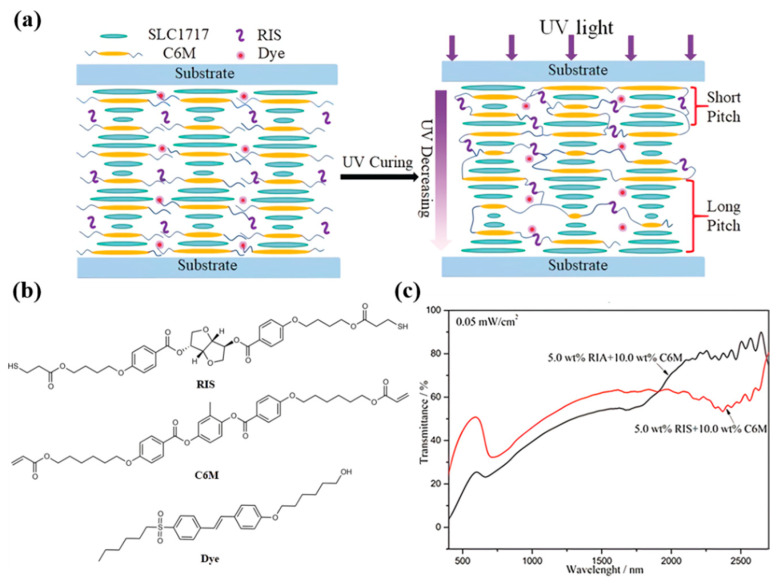
(**a**) Schematic mechanism of the fabrication of PSCLC film with broadband reflection. (**b**) Materials used in this system. (**c**) Transmission spectra of the samples [41]. Reproduced with permission from Hu W, Angew. Chem. Int. Ed.; published by John Wiley and Sons, 2019.

Based on the mechanism of different polymerization rates to induce pitch gradient, Hu synthesized an isosorbide derivative chiral thiol molecule with double-terminal thiyl functional group (RIS) with high HTP (as shown in Figure 5b), and prepared a PSCLC film with broadband reflection properties by thiol-acrylate click chemistry for the first time. The click reaction polymerizes faster than the free radical polymerization of the acrylic monomer [41]. The chiral compounds RIS containing two sulfhydryl groups and C6M can click chemistry under UV light irradiation, while the remaining C6M that did not undergo a click reaction can polymerize by itself. At the side closer to UV light, the light intensity is stronger and the polymerization rate is faster, so C6M and RIS diffuse to this site with a shorter pitch. Therefore, a pitch gradient is formed in the direction of UV irradiation to achieve broadband reflection. Compared with the acrylate-based ChLC film, this system has a wider reflection bandwidth under the same preparation conditions (as shown in Figure 5).

### 2.4. Thermally Induced Molecular Diffusion

**Figure 6:**
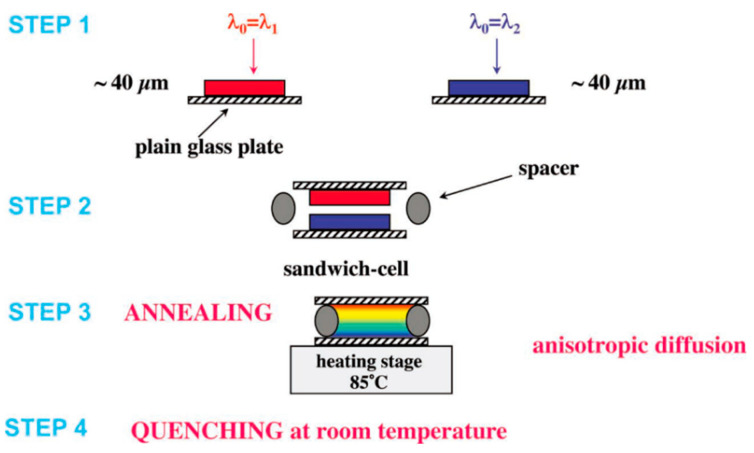
The experimental procedure of thermal diffusion [46]. Reproduced with permission from Mitov M, Adv. Mater.; published by John Wiley and Sons, 2012.

The method of thermally induced molecular diffusion to produce a non-uniform distribution of pitch was first proposed by Mitov [3,42,43,44,45]. Liquid crystal oligomers of cyclosiloxane side chains with different ratios of chiral and non-chiral side chains were stacked together in an up-down way, and thermal diffusion occurred between the two layers after certain heat treatment; then, the films were rapidly cooled to the temperature below the glass transition to fix the gradient distribution of the pitch. Thus, single-layer ChLC polymer films reflecting the whole visible region were obtained (as shown in Figure 6) [46].

**Figure 7:**
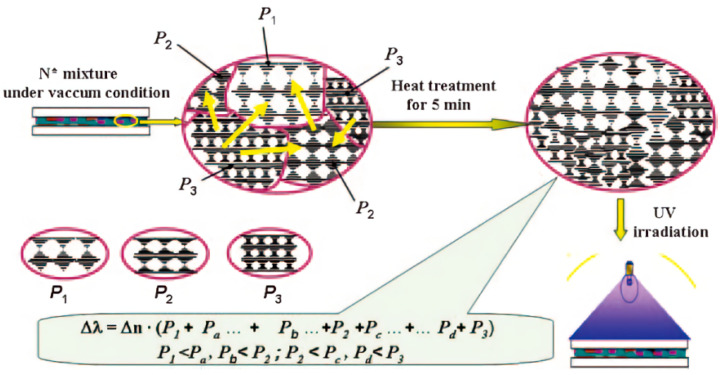
Schematic representation of the preparation of ChLC composite film with random pitch distribution by mixing particles with different pitches. Here, N* in the figure, means the cholesteric phase of ChLC [48]. Reproduced with permission from Huang W, Liq. Cryst.; published by Taylor and Francis, 2008.

This method is also applicable to PSCLC systems [44,45,47]. In this study, the powders with different pitch are randomly mixed in a certain ratio rather than in an up-and-down way. Yang proposed the “powder mixing method”, using glassy cyclosiloxane side chain liquid crystal polymers or polymerizable monomer/chiral compounds with crystalline phase and cholesteric phase transition (as shown in Figure 7). The powders with different pitches were mixed in a certain ratio and heated to liquid crystal phase to achieve the diffusion of material molecules [48]. It is a simple preparation procedure with high controllability of reflection spectrum and bandwidth by adjusting the powder components. On the basis of the above work, Yang mixed chiral dopants of different pitches with photopolymerizable monomers having a crystalline (Cr)- cholesteric phase transition in the solid state and heated them to the ChLC temperature range [49]. The thermal diffusion is accompanied by UV-irradiated polymerization in the parallel orientation state to form monolayer ChLC polymer films with an inhomogeneous pitch distribution structure. 

### 2.5. Two-Phase Coexistence Material System of Cholesteric and Twist Grain Boundary (TGB)

**Figure 8:**
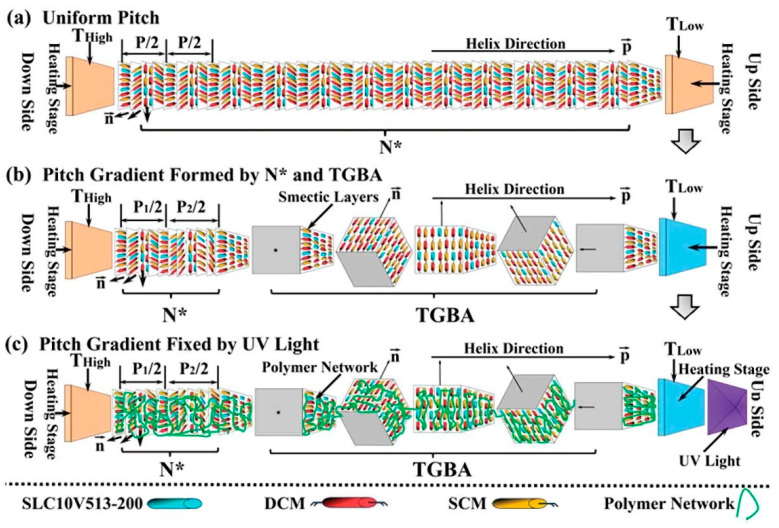
The schematic representation of the mechanism. Here, N* in the figure, means the cholesteric phase of ChLC [50]. Reproduced with permission from He W L, Liq. Cryst.; published by Taylor and Francis, 2017.

**Figure 9:**
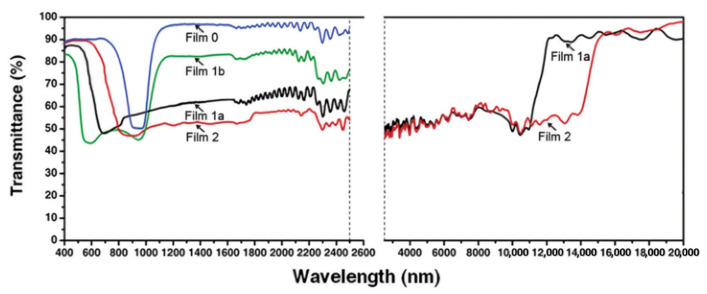
Transmission spectra of Films [51]. Reproduced with permission from Zhang L P, Liq. Cryst.; published by Taylor and Francis, 2016.

Limited by the stability of the helical structure and the average refractive index of the liquid crystal material, it is difficult to form stable broadband reflection for ChLCs with wavelengths over 2000 nm, which severely limits its application in infrared light shielding covering the near-infrared, mid-infrared, and far-infrared wavelengths. To further broaden the reflection bandwidth, based on our preliminary work, we constructed multi-layered microstructures with both cholesteric phase and TGB phase in the ChLC polymerizable material system and achieved a polymeric liquid crystal film with an ultra-wide reflection band [50]. This material system can form an SmA-like short-range ordering (SSO) structure during the transition from cholesteric phase to SmA phase, also called TGB phase, which has a larger pitch than the cholesteric phase and selective light reflection properties. Based on light-controllable polymerization rate and thermally induced molecular diffusion, the liquid crystal cassette was subjected to temperature gradient and light intensity gradient. The liquid crystals were transformed from cholesteric phase helical structure to SSO structure in the cell thickness direction, and polymer films with both cholesteric phase and TGB phase could be fixed after UV polymerization (as shown in Figure 8). Microstructure observation by scanning electron microscopy showed that the pitch of the film had an ultra-wide distribution, and the reflection spectrum of the film could cover 750–2500 nm. Similarly, Zhang prepared ultra-wide infrared reflective films by controlling the curing temperature and utilizing the difference in photopolymerization rates of different acrylate monomers and mesocrystalline phase structure transformation [51]. Due to the higher polymerization rate of the diacrylate monomer than the monoacrylate monomer, a gradient in pitch distribution is created in the thick direction of the liquid crystal cell with both cholesteric phase helical structure and SSO structure. The film reflects light in a wavelength range from 780 nm to 14,000 nm and transmits visible light, as shown in Figure 9.

### 2.6. Memory Effects of the Template

In order to achieve more effective and practical modulation, a two-step approach called “wash-out/refill” can be used to prepare PSCLC films with broadband reflection [52,53,54,55,56,57]. First, a polymer network template of a certain pitch is obtained by removing low-molar-mass ChLC, and then another ChLC of a different pitch is introduced. The ChLC with broadband reflection is achieved by using the memory effect of the helical structure of the polymer network template [53].

**Figure 10:**
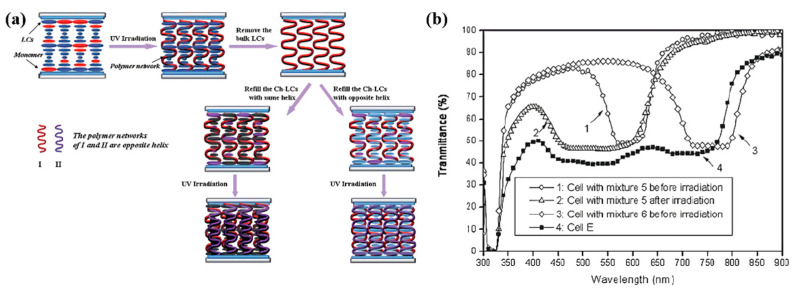
(**a**) The procedure for preparing the ChLC film. (**b**) The transmission spectra of a cell with mixture 5 before and after UV irradiation, a cell with mixture 6, and cell E [58]. Reproduced with permission from Guo J B, J. Mater. Chem.; published by Royal Society of Chemistry, 2010.

Guo used ChLCs with different pitches in ChLCs/polymer template composites by a wash-out/filling method, which enables simultaneous red, green, and blue reflections and broadband reflections in monolayer ChLC films (as shown in Figure 10). Moreover, the temperature-tunable photonic bandgap of ChLC films was obtained by filling the polymer template with thermally sensitive hydrogen-bonded self-assembled ChLCs. The bandwidth of the cell E can reach 340 nm (450–790 nm) due to the superposition of two reflection bands of the above ChLC mixtures 5 and 6, which includes red, green and blue lights in the visible light spectra [58]. This demonstrates that the helix-structure memory of the polymer template, which is derived from the initial PSCLC, plays an important role. Similarly, Shi used a photo-induced molecular diffusion method by adding ultraviolet (UV) absorbing dye to the liquid crystal system and washing away the unreacted liquid crystal monomers after polymerization with UV light. The polymer network shrank in the absence of liquid crystal support, leading to a reduction in pitch. Refilling the liquid crystal SLC-1717, the sample still had the effect of broadband reflection due to the memory effect of the polymer network [59]. Zhang studied the performance of the polymer network during the wash-out/refilling process and the effect of monomer concentration and polymerization conditions on the polymer network. Due to the shrinkage of the polymer network and the shift in refractive index after rinsing, the reflection band was blue-shifted compared to the original reflective band. The microstructure of the polymer network was an important factor affecting the performance of the polymer network. Dense polymer networks with small pores exhibited better memory effects on the reflection band. As the light intensity, monomer concentration, and polymerization time increase, the polymer network will better reproduce the reflection band [60].

**Figure 11:**
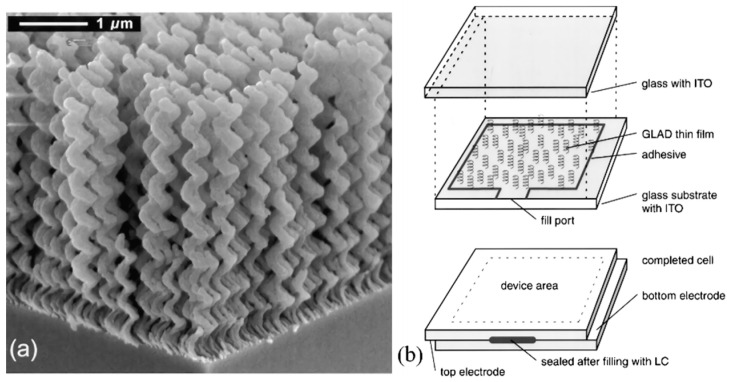
(**a**) Examples of helical GLAD films (by SEM). (**b**) Structure of a sandwich cell with a porous chiral film [63]. Reproduced with permission from Sit J C, Liq. Cryst.; published by Taylor and Francis, 2000.

Robbie demonstrated that inorganic film templates with porous structures can also induce the orientation of liquid crystals and form pitch gradients [61]. Porous inorganic networks of SiO2 helical columns with pitch linearly graded from 350 nm (at substrate) to 500 nm (top of the film) were deposited onto glass substrates by using a grazing angle deposition (GLAD) technique [62]. By permeating nematic phase liquid crystals into this porous film, the composite film exhibited optical properties similar to those of a broadband reflection ChLC film with a helically twisted structure of pitch gradient (as shown in Figure 11) [63]. Wang obtained a silica aerogel film/chiral nematic liquid crystal composite film by coating one side surface of the cell with silica aerogel film and the other side surface of the cell with polyvinyl alcohol (PVA) after orientation, then filling the cell with ChLCs. As the porosity of the silica aerogel film increases, the reflection bandwidth of the composite film increases [64]. Zhao prepared ZnO nanorod arrays with high aspect ratios at different growth times by hydrothermal reaction on one side of the cell and then filled the cell with ChLCs. It was shown that the gap of nanorods accommodates small-pitch ChLCs and affects the cross-linking motion of polymerizable monomers. The reflection broadband can be manipulated by the length of the ZnO nanorods [65].

### 2.7. Electromagnetic-Field-Induced Molecular Diffusion

**Figure 12:**
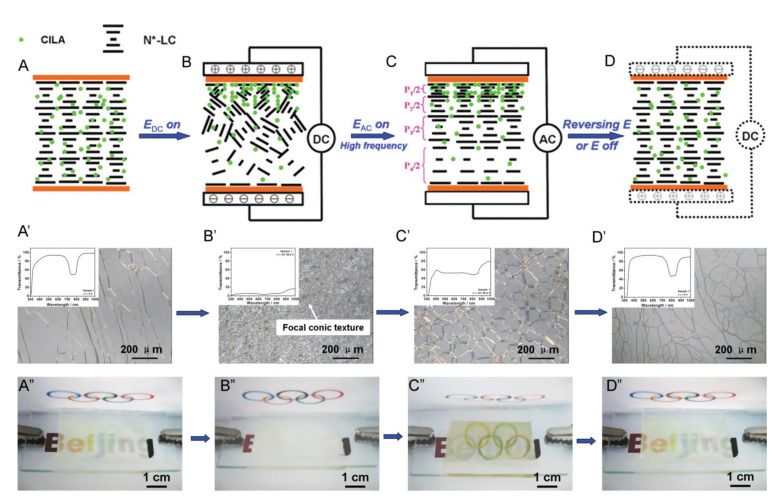
(**A**–**D**) Schematic representation of the molecular arrangements at different electric fields, (**A’**–**D’**) Microscopy images of the textures observed under POM, and (**A’’**–**D’’**) Photos of different electric-field-induced states [67]. Reproduced with permission from Hu W, Adv. Mater.; published by John Wiley and Sons, 2010.

The electromagnetic field induces molecular diffusion mainly by linking particles responding to an electromagnetic field to chiral groups through covalent or non-covalent bonds or electrostatic forces. The movement of these particles is controlled by the electromagnetic field to induce the movement of the chiral groups, resulting in a difference in the chiral concentration of the system and causing a gradient in the pitch [66]. As shown in Figure 12, Yang added anionic chiral ionic liquids containing chiral groups to the ChLC material system. Under the action of an applied high-frequency AC electric field, the anion in the ionic liquid moves toward the positive pole and causes a higher concentration of the chiral group near the positive pole and a lower concentration near the negative pole, resulting in a concentration gradient of the chiral compound. When the applied voltage reaches 40 V, the reflected wavelength can cover the entire visible wavelength band [67].

**Figure 13:**
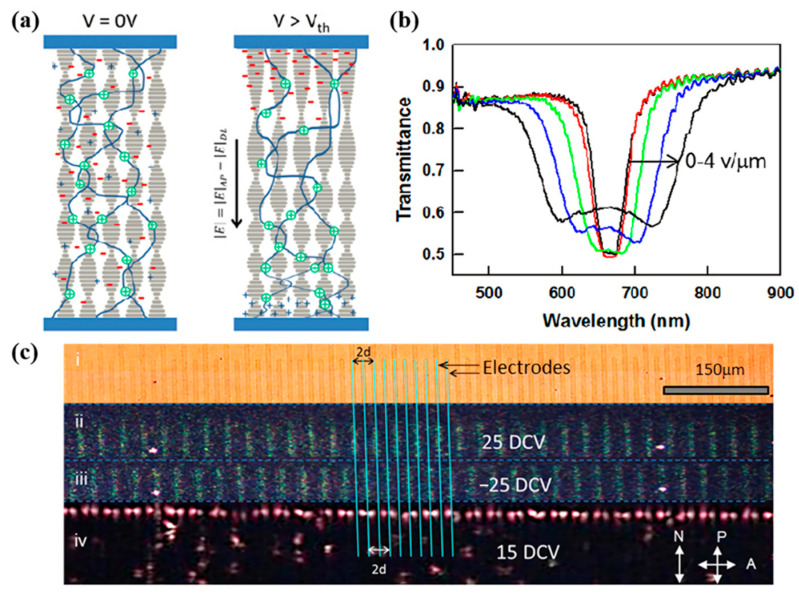
(**a**) Illustration of the ionic-charge-trapping mechanism proposed. (**b**) Transmission spectra of a PSCLC cell subjected to DC fields ranging from 0 to 4 V/μm. (**c**) Transmission micrographs of cells prepared with patterned (interdigitated) electrodes [74]. Reprinted under the Creative Commons Attribution License (CCAL) terms.

In particular, in the PSCLC system, it was found that the application of a DC electric field induced a significant broadening of the reflection bandwidth and returned to the initial state after the removal of the electric field [68,69]. The studies show that liquid crystal mixtures usually contain a certain amount of ionic hybrids (usually 10^9^ to 10^14^/cm^3^) due to the chemical synthesis process and the use of orientation agents, photoinitiators, and polymerizable monomers [70,71,72]. The polymerizable monomers of acrylates can effectively adsorb cations during the polymerization reaction, so the polymer network will be positively charged due to the selective adsorption of cations, and the network can move directionally by the electric field force under the action of a DC electric field [66,73]. The material system is made to undergo pitch expansion near the positive electrode and pitch compression near the negative electrode, thus, creating a pitch gradient within the cell [74,75,76]. The dominant role of structural chirality and the effect of cross-linking of polymer-stabilized networks on the relative changes in threshold voltage and bandwidth per-voltage were further investigated (as shown in Figure 13). It is important to control the polymer network morphology and ion density, which, in turn, determined the reflection bandwidth [77,78,79]. Based on fluorescent-molecule-doped PSCLC, Li successfully fabricated electric-field-driven systems with tunable circularly polarized luminescence signals. The applied electric field can expand the reflection bandwidth of PSCLC from 100 nm to 350 nm, covering the entire visible region [80]. Zhang used the semiconductor material poly(N-vinylcarbazole) (PVK) as the orientation layer of PSCLC. Under UV irradiation, the PVK layer generated hole-electron pairs that neutralized the impurity electrons in the LC-PVK junction, resulting in a lower built-in electric field in the LC device, which was more favorable for the polymer network to move under the electric field. Compared with the PVA orientation layer, the bandwidth broadening of the PVK orientation layer was better at the same electric field (up to 821 nm at 60 V) [81].

**Figure 14:**
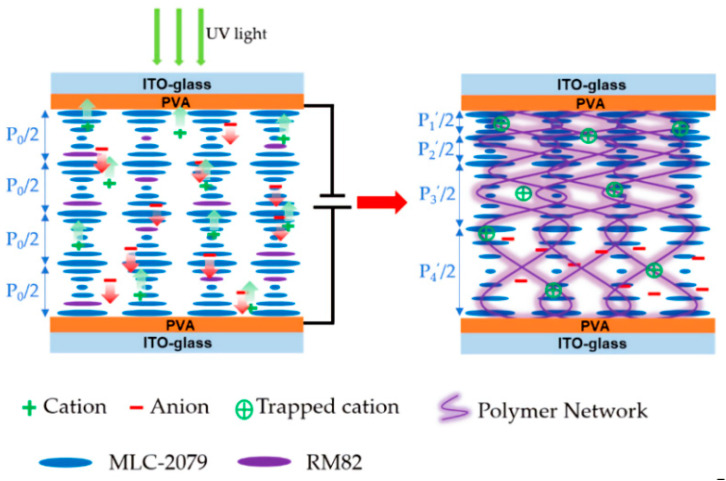
Schematic illustration of pitch-length modulation in ChLC cell [82]. Reprinted under the Creative Commons Attribution License (CCAL) terms.

Based on the ion capture of oligomers, Hu developed an in-situ DC curing strategy, which involves UV irradiation while loading DC voltage (as shown in Figure 14). During the slow polymerization process, the oligomers formed first are able to trap impurity cations. Under in-situ DC bias, these trapped cations drag the oligomers toward the cathode, where they form short pitches and are further immobilized under UV light irradiation. Thus, oligomer drift and further polymerization occur simultaneously, resulting in a final nonuniform network that modulates the pitch length of the ChLC over a larger range [82].

**Figure 15:**
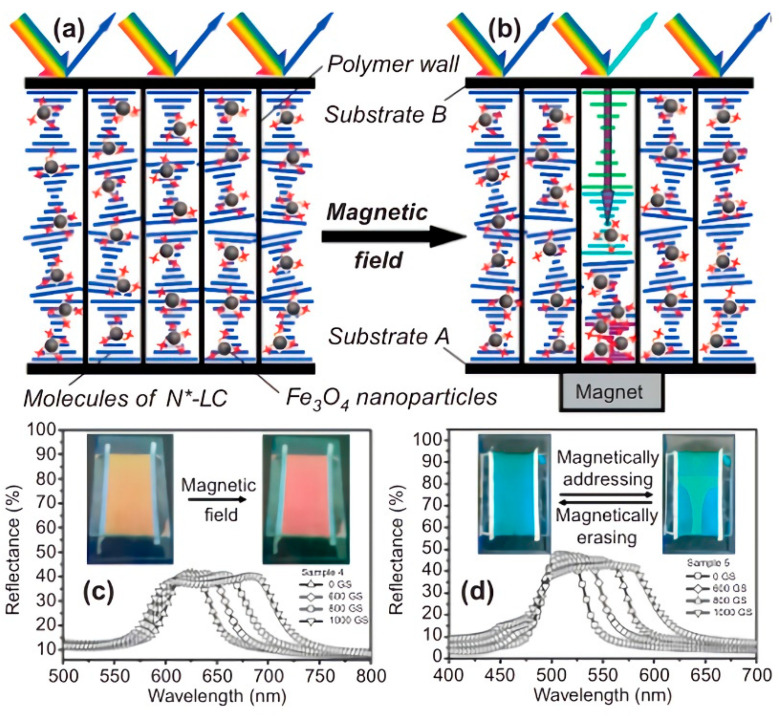
(**a**,**b**) The schematic mechanism of magnetic-field-induced broadband reflection (**c**,**d**) the reflection spectra of samples 4 and 5 at different magnetic field strengths [83]. Reproduced with permission from Hu W, Liq. Cryst.; published by Taylor and Francis, 2010.

In the field of magnetic field induction, Yang modified magnetic nanoparticles Fe_3_O_4_ to make the remaining -OH groups on the surface of the particles self-assemble with chiral compounds containing pyridine groups to form hydrogen bonds. When a magnetic field was applied at a certain position of the sample, the magnetic nanoparticles could aggregate to that position while the chiral compounds follow the magnetic particles due to hydrogen bonding. A concentration gradient of chiral compounds was formed in the direction of the cell thickness, resulting in a pitch gradient distribution (as shown in Figure 15). The material system has magnetic writing and magnetic erasure properties and has a good application in the field of reflective color displays [83]. 

## 3. The Application of ChLCs with Broadband Reflection Properties

### 3.1. Multilayer System

Dye-sensitized solar cells are a type of solar cell made from low-cost nanoporous semiconductor films and light-sensitive dyes as the main raw material and have received a lot of attention in recent years [84]. However, the power conversion efficiency of dye-sensitized solar cells is relatively low compared with that of general solar cells. Therefore, the main research direction at this stage is to improve power conversion. Increasing the absorption spectrum range of the cell has become a way to enhance conversion efficiency [85]. The use of broadband reflective ChLCs as battery-back reflectors allows the battery to capture more sunlight and improves the light absorption in the battery. 

Liu demonstrated a simple strategy to enhance the light absorption of devices by using a ChLC polymer film with broadband reflection as transparent and flexible back reflectors for dye-sensitized solar cells. The pitch and twist direction of the ChLC polymer films are precisely controlled by modulating the type and concentration of the chiral dopant to produce a selective reflection of light in a specific wavelength range. These ChLC polymer films dramatically improve the light absorption of the device and maintain the transparency of the dye-sensitized solar cell. This method allows the photonic bandgap of the ChLC back reflector to be broadened and tuned to a spectral range suitable for the absorption of dye molecules, thus, improving the light-capture capability of the dye-sensitized solar cell [86].

### 3.2. Efficient Infrared Shielding Films

At present, building energy consumption accounts for more than 30% of the total social energy consumption, and windows are the key link affecting building energy consumption. In modern buildings, glass windows play an important role in making the built environment more comfortable and sustainable [87,88]. Infrared radiation at wavelengths of 800–2000 nm is reported to account for more than 50% of the entire solar infrared radiation energy [89]. Infrared-adjustable smart windows can effectively regulate the incidence and reflection of infrared light, and they are the primary choice for realizing smart buildings.

Theoretically, if the reflection band in the designed ChLC material is in the near-infrared region, so it can be used as an infrared shielding film. Of course, it is necessary to superimpose left-handed and right-handed ChLC films in order to break through the shielding of only 50% of the infrared light incident from the outdoors. The prepared infrared reflectors with wide reflection bands have sufficient flexibility under mechanical deformation and high transparency in the visible spectrum by superimposing ChLC films with different spin directions on PET substrates [90]. At present, the infrared reflection bandwidth of the prepared PSCLC is fixed and cannot be adjusted, so its practicability is reduced. Further, if the designed ChLC materials have sensitive groups or microstructures that respond to stimuli, the prepared infrared shielding film will have the characteristics of responding to external stimuli. Khandelwal adjusted the reflection bandwidth of the optical switch by applying DC voltage, that is, making the infrared shielding film have the characteristics of electrical tuning. Tunable infrared reflective films were fabricated by using a PSCLC containing negative dielectric, ethylene glycol double crosslinker, and negative liquid crystal. Due to the use of a special photopolymerization monomer and ethylene glycol double crosslinker, the polymer network formed after polymerization under UV light can capture more cations. When a DC electric field is applied, the polymer network is charged by capturing polar cations through electrostatic force. The polymer network moves under the action of electrostatic force, resulting in the compression of the pitch of CLC on the negative electrode and the extension on the other side, thus, forming a pitch gradient and obtaining a reflection bandwidth from 700 nm to 1800 nm. When no electric field is applied, the spacing of CLC is evenly distributed in the whole system, and narrow-band reflection can be generated at this time [91]. Similarly, Hu developed an electrically tunable infrared (IR) reflector based on PSCLC, which can reflect broadband IR light from 725 nm to 1435 nm when a DC electric field is applied, while transmitting at more than 90% in the visible region. Model experiments show that this IR reflector can effectively control the room temperature compared to ordinary glass [92].

**Figure 16:**
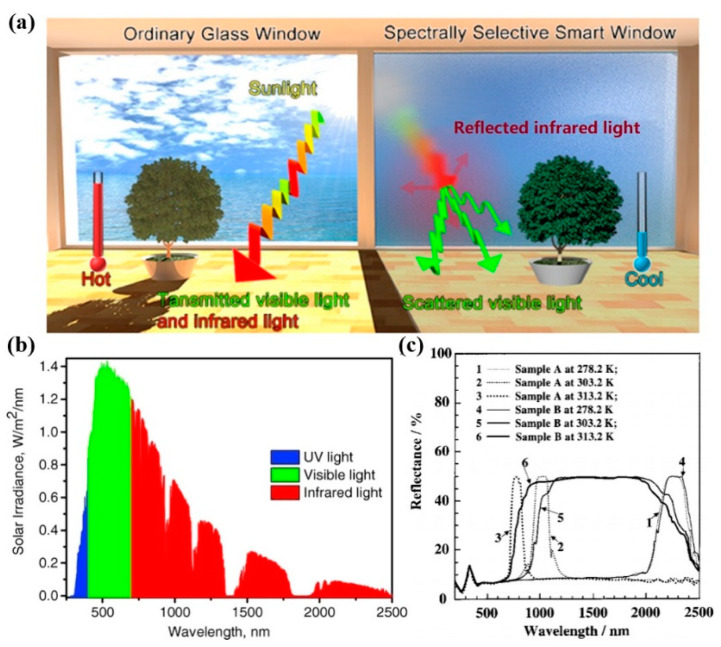
(**a**) The effect of infrared shielding smart window illustration [87]. Reproduced with permission from Wu M, ACS Appl. Mater. Interfaces; published by ACS, 2018. (**b**) Solar spectrum on Earth [89]. Reproduced with permission from Khandelwal H, Adv. Energy Mater; published by John Wiley and Sons, 2017. (**c**) Selective reflection spectra of samples A and B measured at different temperatures [93]. Reproduced with permission from Yang H, Appl. Phys. Lett.; published by AIP Publishing, 2003.

Yang doped ChLC materials with temperature-responsive chiral materials and developed an effective temperature-responsive smart window [93]. Due to the fact that the HTP of the chiral molecule increases with an increase in temperature, the liquid crystal material obtained by doping temperature-responsive chiral materials reflects infrared light in a range of 2050–2400 nm at a lower temperature (about 5 °C). When the temperature rose to 40 °C and 50 °C, it would reflect infrared light in a range of 950–2400 nm and 800–2400 nm, respectively, as shown in Figure 16c. This infrared reflector allowed the most infrared light to enter the room in winter and could reflect a large amount of infrared energy from sunlight in summer, thus, saving energy. Zhang prepared a temperature-responsive infrared reflective coating consisting of PSCLC siloxanes by a simple rod-coating technique. The reflection band of the PSCLC coating was continuously blue shifted from 1400 nm to 800 nm when the temperature increased from room temperature [94]. In general, it is still a challenge to further expand the reflection range of infrared light and the regulation range of stimulus response when ChLCs are used in infrared light shielding windows.

### 3.3. Laser Protection Films

With the continuous development of laser technology, lasers have been widely used in medical, welding, and other fields. Since lasers can cause damage to human eyes and optical devices of equipment, laser protection has become one of the hot research topics of the day. Traditional laser protection materials have the problem of single-wavelength protection and it is necessary to develop materials with a wide range of protection [95].

Zhao prepared a novel composite film of ChLCs doped with the efficient light-limiting material 6, 6-phenyl C61 butyric acid methyl ester by UV polymerization. ChLCs have the desired reflection wavelength covering from 750 nm to 2300 nm and can effectively prevent continuous or tunable laser light in the near-infrared (NIR) region. The composite film exhibits not only broadband reflection in the NIR region, but also strong nonlinear absorption and excellent optical limiting properties in the visible region, demonstrating the potential application of the film for laser damage protection [96].

## 4. Conclusions and Outlook

ChLCs with broadband reflection characteristics have outstanding attraction and wide market requirements in both scientific research and practical industrialization. This paper reviews the methods for broadening the reflectance bandwidth of ChLCs in terms of a multilayer system, responsive chiral molecules, thermal induction, optical induction, electromagnetic induction, two-phase coexistence system, and template memory effects. The advantages of the multilayer system method are a simple preparation process and controllable reflection wavelength range, but there is the problem of interfacial diffusion. The reflection bandwidth of ChLCs can be further broadened by changing the concentration distribution of chiral molecules directly or indirectly, by using the change in HTP value of responsive chiral molecules with external environment, or by combining various methods. The diffusion of chiral molecules induced by external environmental stimuli can be controlled by modulating the influencing factors, such as light intensity, electromagnetic field strength, monomer type and content, and so on, to regulate the reflection bandwidth and range. In conclusion, ChLCs with broadband reflection can be achieved by constructing self-organized nanostructures with a pitch gradient or non-uniform pitch distribution. Due to their excellent performance, ChLCs with broadband reflection have a wide application market in dye-sensitized solar cells, infrared shielding smart windows, laser protection films, etc. However, it is still a major challenge to achieve its simple and large-scale industrial preparation and to overcome the reflectance limitation. Future research needs to focus on the development of new material systems and developing universal and feasible band-broadening processes. 

## Data Availability

Not applicable.

## Sample Availability

Samples of the compounds are not available from the authors.

## References

[1] Tamaoki N. (2001). Cholesteric liquid crystals for color information technology. Adv. Mater..

[2] Broer D.J., Lub J., Mol G.N. (1997). Photo-controlled diffusion in reacting liquid crystals: A new tool for the creation of complex molecular architectures. Macromol. Symp..

[3] Boudet A., Binet C., Mitov M., Bourgerette C., Boucher E. (2000). Microstructure of variable pitch cholesteric films and its relationship with the optical properties. Eur. Phys. J. E.

[4] Ha N.Y., Ohtsuka Y., Jeong S.M., Nishimura S., Suzaki G., Takanishi Y., Ishikawa K., Takezoe H. (2008). Fabrication of a simultaneous red-green-blue reflector using single-pitched cholesteric liquid crystals. Nat. Mater..

[5] Bisoyi H.K., Bunning T.J., Li Q. (2018). Stimuli-Driven Control of the Helical Axis of Self-Organized Soft Helical Superstructures. Adv. Mater..

[6] Belyakov V., Dmitrienko V.E., Orlov V. (1979). Optics of cholesteric liquid crystals. Sov. Phys. Usp..

[7] Meyer R.B. (1968). Effects of electric and magnetic fields on the structusre of cholesteric liquid crystals. Appl. Phys. Lett..

[8] Dreher R., Meier G., Saupe A. (1971). Selective reflection by cholesteric liquid crystals. Mol. Cryst. Liq. Cryst..

[9] Dreher R., Meier G. (1973). Optical properties of cholesteric liquid crystals. Phys. Rev. A.

[10] Saeva F., Wysocki J.J. (1971). Induced circular dichroism in cholesteric liquid crystals. J. Am. Chem. Soc..

[11] Sackmann E., Voss J. (1972). Circular dichroism of helically arranged molecules in cholesteric phases. Chem. Phys. Lett..

[12] Day G., Gaddy O. (1968). Electric-field-induced optical rotation in cholesteric liquid crystals. Proc. IEEE.

[13] Gerritsen H.J., Yamaguchi R.T. (1971). A microwave analog of optical rotation in cholesteric liquid crystals. Am. J. Phys..

[14] Smith C., Sabatino D., Praisner T. (2001). Temperature sensing with thermochromic liquid crystals. Exp. Fluids.

[15] Tagaya A., Ishii S., Yokoyama K., Higuchi E., Koike Y. (2002). The advanced highly scattering optical transmission polymer backlight for liquid crystal displays. Jpn. J. Appl. Phys..

[16] Steinsträsser R., Pohl L. (1973). Chemistry and applications of liquid crystals. Angew. Chem. Int. Ed. Engl..

[17] Ilchishin I., Tikhonov E.A., Tishchenko V., Shpak M. (1980). Generation of a tunable radiation by impurity cholesteric liquid crystals. Jetp Lett..

[18] Kopp V., Fan B., Vithana H., Genack A. (1998). Low-threshold lasing at the edge of a photonic stop band in cholesteric liquid crystals. Opt. Lett..

[19] Binet C., Mitov M., Mauzac M. (2001). Switchable broadband light reflection in polymer-stabilized cholesteric liquid crystals. J. Appl. Phys..

[20] Bin Y., Lu Q. (2003). Status of research and application of multifunctional stealth materials. Infrared Technol..

[21] Mitov M., Dessaud N. (2006). Going beyond the reflectance limit of cholesteric liquid crystals. Nat. Mater..

[22] Gauza S., Wen C.-H., Wu S.-T., Janarthanan N., Hsu C.-S. (2004). Super high birefringence isothiocyanato biphenyl-bistolane liquid crystals. Jpn. J. Appl. Phys..

[23] Gauza S., Wang H., Wen C.-H., Wu S.-T., Seed A.J., Roman D. (2003). High birefringence isothiocyanato tolane liquid crystals. Jpn. J. Appl. Phys..

[24] White T.J., Broer D.J. (2015). Programmable and adaptive mechanics with liquid crystal polymer networks and elastomers. Nat. Mater..

[25] Herbert K.M., Fowler H.E., McCracken J.M., Schlafmann K.R., Koch J.A., White T.J. (2022). Synthesis and alignment of liquid crystalline elastomers. Nat. Rev. Mater.

[26] Liang X., Guo S., Chen M., Li C., Wang Q., Zou C., Zhang C., Zhang L., Guo S., Yang H. (2017). A temperature and electric field-responsive flexible smart film with full broadband optical modulation. Mater. Horiz..

[27] Kralik J.C., Fan B., Vithana H., Li L., Faris S.M. (1997). Backlight output enhancement using cholesteric liquid crystal films. Mol. Cryst. Liq. Cryst. Sci. Technol. Sect. A-Mol. Cryst. Liq. Cryst..

[28] Choi H., Kim J., Nishimura S., Toyooka T., Araoka F., Ishikawa K., Wu J.W., Takezoe H. (2010). Broadband Cavity-Mode Lasing from Dye-Doped Nematic Liquid Crystals Sandwiched by Broadband Cholesteric Liquid Crystal Bragg Reflectors. Adv. Mater..

[29] He W., Yao D., Luo S., Xiong R., Yuan X. (2022). Broadband Reflective Liquid Crystal Films Prepared by Rapid Inkjet Printing and Superposition Polymerization. Crystals.

[30] Guo R.W., Li K.X., Cao H., Wu X.J., Wang G.J., Cheng Z.H., Wang F.F., Zhang H.Q., Yang H.A. (2010). Chiral polymer networks with a broad reflection band achieved with varying temperature. Polymer.

[31] Duan M.Y., Cao H., Wu Y., Li E.L., Wang H.H., Wang D., Yang Z., He W.L., Yang H. (2017). Broadband reflection in polymer stabilized cholesteric liquid crystal films with stepwise photo-polymerization. Phys. Chem. Chem. Phys..

[32] Zhang D.D., Cao H., Duan M.Y., Wang H.H., Chen Y.J., Zong C., Gan P., Zhao L.M., Yang Z., Wang D. (2019). Effect of Monomer Composition on the Performance of Polymer-Stabilized Liquid Crystals with Two-Step Photopolymerization. J. Polym. Sci. Part B Polym. Phys..

[33] Chen X.W., Wang L., Chen Y.J., Li C.Y., Hou G.Y., Liu X., Zhang X.G., He W.L., Yang H. (2014). Broadband reflection of polymer-stabilized chiral nematic liquid crystals induced by a chiral azobenzene compound. Chem. Commun..

[34] Lu H.B., Xie X.Y., Xing J., Xu C., Wu Z.Q., Zhang G.B., Lv G.Q., Qiu L.Z. (2016). Wavelength-tuning and band-broadening of a cholesteric liquid crystal induced by a cyclic chiral azobenzene compound. Opt. Mater. Express.

[35] Sun J., Yu L., Wang L., Li C., Yang Z., He W., Zhang C., Zhang L., Xiao J., Yuan X. (2017). Optical intensity-driven reversible photonic bandgaps in self-organized helical superstructures with handedness inversion. J. Mater. Chem. C.

[36] Gan P., Zhang X., Zhao L., Shi W., Cao H., Wang H., Yang Z., Wang D., He W. (2021). Broadband reflection in polymer-stabilized cholesteric liquid crystal film with zinc oxide nanoparticles film thermal diffusion method. Liq. Cryst..

[37] Muraveva V., Kozmik V., Kohout M., Manko A., Piryazev A., Ivanov D., Abramchuk S., Cigl M., Bobrovsky A. (2022). The smectogenity as a crucial factor of broadening of the selective light reflection peak in cholesteric photopolymerizable mixtures. Liq. Cryst..

[38] Broer D.J., Lub J., Mol G.N. (1995). Wide-band reflective polarizers from cholesteric polymer networks with a pitch gradient. Nature.

[39] Zhang Y.-S., Emelyanenko A.V., Liu J.-H. (2017). Fabrication and optical characterization of imprinted broad-band photonic films via multiple gradient UV photopolymerization. J. Polym. Sci. Part B Polym. Phys..

[40] Relaix S., Bourgerette C., Mitov M. (2007). Broadband reflective cholesteric liquid crystalline gels: Volume distribution of reflection properties and polymer network in relation with the geometry of the cell photopolymerization. Liq. Cryst..

[41] Hu W., Chen M., Wang Q., Zhang L.Y., Yuan X.T., Chen F.W., Yang H. (2019). Broadband Reflection in Polymer-Stabilized Cholesteric Liquid Crystals via Thiol-Acrylate Chemistry. Angew. Chem. Int. Ed..

[42] Mitov M., Boudet A., Sopéna P. (1999). From selective to wide-band light reflection: A simple thermal diffusion in a glassy cholesteric liquid crystal. Eur. Phys. J. B.

[43] Binet C., Mitov M., Boudet A. (2000). Bragg reflections in cholesteric liquid crystals: From selectivity to broadening and reciprocally. Mol. Cryst. Liq. Cryst. Sci. Technol. Sect. A-Mol. Cryst. Liq. Cryst..

[44] Nouvet E., Mitov M. (2004). New optical memory effects in polymer-stabilized cholesteric liquid crystals due to pitch changes during the UV-curing. Mol. Cryst. Liq. Cryst..

[45] Zografopoulos D.C., Kriezis E.E., Mitov M., Binet C. (2006). Theoretical and experimental optical studies of cholesteric liquid crystal films with thermally induced pitch gradients. Phys. Rev. E.

[46] Mitov M. (2012). Cholesteric Liquid Crystals with a Broad Light Reflection Band. Adv. Mater..

[47] Wang F.F., Li K.X., Song P., Wu X.J., Chen H.P., Cao H. (2013). The effects of thermally induced diffusion of dye on the broadband reflection performance of cholesteric liquid crystals films. Compos. Pt. B-Eng..

[48] Huang W., Bian Z.Y., Li K.X., Xiao J.M., Cao H., Yang H. (2008). Study on selective reflection properties of chiral nematic liquid crystalline composites with a non-uniform pitch distribution. Liq. Cryst..

[49] Bian Z.Y., Li K.X., Huang W., Cao H., Yang H., Zhang H.Q. (2007). Characteristics of selective reflection of chiral nematic liquid crystalline gels with a nonuniform pitch distribution. Appl. Phys. Lett..

[50] He W.L., Wang F.F., Song P., Li K.X., Ding H.J., Yang Z., Xiao J.M., Li F.S. (2016). Broadband reflective liquid crystal films induced by facile temperature-dependent coexistence of chiral nematic and TGB phase. Liq. Cryst..

[51] Zhang L.P., Wang M., Wang L., Yang D.K., Yu H.F., Yang H. (2016). Polymeric infrared reflective thin films with ultra-broad bandwidth. Liq. Cryst..

[52] Guo J.B., Yang H., Li R., Ji N., Dong X.M., Wu H., Wei J. (2009). Effect of Network Concentration on the Performance of Polymer-Stabilized Cholesteric Liquid Crystals with a Double-Handed Circularly Polarized Light Reflection Band. J. Phys. Chem. C.

[53] Guo J.B., Liu F., Chen F.J., Wei J., Yang H. (2010). Realisation of cholesteric liquid-crystalline materials reflecting both right- and left-circularly polarised light using the wash-out/refill technique. Liq. Cryst..

[54] Lin J.D., Chu C.L., Lin H.Y., You B.W., Horng C.T., Huang S.Y., Mo T.S., Huang C.Y., Lee C.R. (2015). Wide-band tunable photonic bandgaps based on nematic-refilling cholesteric liquid crystal polymer template samples. Opt. Mater. Express.

[55] Zhao Y.Z., Zhang L.Y., He Z.M., Chen G., Wang D., Zhang H.Q., Yang H. (2015). Photoinduced polymer-stabilised chiral nematic liquid crystal films reflecting both right- and left-circularly polarised light. Liq. Cryst..

[56] McConney M.E., Tondiglia V.P., Hurtubise J.M., White T.J., Bunning T.J. (2011). Photoinduced hyper-reflective cholesteric liquid crystals enabled via surface initiated photopolymerization. Chem. Commun..

[57] Choi S.S., Morris S.M., Huck W.T.S., Coles H.J. (2010). Simultaneous red-green-blue reflection and wavelength tuning from an achiral liquid crystal and a polymer template. Adv. Mater..

[58] Guo J.B., Wu H., Chen F.J., Zhang L.P., He W.L., Yang H., Wei J. (2010). Fabrication of multi-pitched photonic structure in cholesteric liquid crystals based on a polymer template with helical structure. J. Mater. Chem..

[59] Shi W.T., Zhang X.T., Han R., Li H., Cao H., Chen Y.J., Wang D., Yang Z., He W.L. (2021). Preparation of cholesteric polymer networks with broadband reflection memory effect. Liq. Cryst..

[60] Zhang D., Shi W., Cao H., Chen Y., Zhao L., Gan P., Yang Z., Wang D., He W. (2020). Reflective Band Memory Effect of Cholesteric Polymer Networks Based on Washout/Refilling Method. Macromol. Chem. Phys..

[61] Robbie K., Broer D.J., Brett M.J. (1999). Chiral nematic order in liquid crystals imposed by an engineered inorganic nanostructure. Nature.

[62] Robbie K., Sit J.C., Brett M.J. (1998). Advanced Techniques for Glancing Angle Deposition. J. Vac. Sci. Technol. B.

[63] Sit J.C., Broer D.J., Brett M.J. (2000). Alignment and switching of nematic liquid crystals embedded in porous chiral thin films. Liq. Cryst..

[64] Wang X.B., Zhang Y., Luo J.Y., Wang D., Gao H., Zhang J.J., Xing Y., Yang Z., Cao H., He W.L. (2018). Silica aerogel films via ambient pressure drying for broadband reflectors. New J. Chem..

[65] Zhao Y.Z., He Z.M., Zhang H.M., Zhao Y., Li K.X., Zhang Y.M., Miao Z.C. (2020). Preparation and properties of ZnO nanorod array/liquid crystal composite system. Liq. Cryst..

[66] Hu W., Zhang L.P., Cao H., Song L., Zhao H.Y., Yang Z., Cheng Z.H., Yang H.A., Guo L. (2010). Electro-optical study of chiral nematic liquid crystal/chiral ionic liquid composites with electrically controllable selective reflection characteristics. Phys. Chem. Chem. Phys..

[67] Hu W., Zhao H.Y., Song L., Yang Z., Cao H., Cheng Z.H., Liu Q., Yang H. (2010). Electrically Controllable Selective Reflection of Chiral Nematic Liquid Crystal/Chiral Ionic Liquid Composites. Adv. Mater..

[68] Tondiglia V.T., Natarajan L.V., Bailey C.A., Duning M.M., Sutherland R.L., Ke-Yang D., Voevodin A., White T.J., Bunning T.J. (2011). Electrically induced bandwidth broadening in polymer stabilized cholesteric liquid crystals. J. Appl. Phys..

[69] Lu H.B., Wei C., Zhang Q., Xu M., Ding Y.S., Zhang G.B., Zhu J., Xie K., Zhang X.J., Hu Z.J. (2019). Wide tunable laser based on electrically regulated bandwidth broadening in polymer-stabilized cholesteric liquid crystal. Photonics Res..

[70] Chen H.Y., Yang K.X., Lee W. (2004). Transient behavior of the polarity-reversal current in a nematic liquid-crystal device. Opt. Express.

[71] Lee K.M., Tondiglia V.P., White T.J. (2016). Photosensitivity of reflection notch tuning and broadening in polymer stabilized cholesteric liquid crystals. Soft Matter.

[72] Yu M.N., Wang L., Nemati H., Yang H., Bunning T., Yang D.K. (2017). Effects of polymer network on electrically induced reflection band broadening of cholesteric liquid crystals. J. Polym. Sci. Part B Polym. Phys..

[73] Lu L., Sergan V., Bos P.J. (2012). Mechanism of electric-field-induced segregation of additives in a liquid-crystal host. Phys. Rev. E.

[74] Tondiglia V.P., Natarajan L.V., Bailey C.A., McConney M.E., Lee K.M., Bunning T.J., Zola R., Nemati H., Yang D.K., White T.J. (2014). Bandwidth broadening induced by ionic interactions in polymer stabilized cholesteric liquid crystals. Opt. Mater. Express.

[75] Nemati H., Liu S., Zola R.S., Tondiglia V.P., Lee K.M., White T., Bunning T., Yang D.K. (2015). Mechanism of electrically induced photonic band gap broadening in polymer stabilized cholesteric liquid crystals with negative dielectric anisotropies. Soft Matter.

[76] Lee K.M., Tondiglia V.P., Lee T., Smalyukh I.I., White T.J. (2015). Large range electrically-induced reflection notch tuning in polymer stabilized cholesteric liquid crystals. J. Mater. Chem. C.

[77] Lee K.M., Tondiglia V.P., McConney M.E., Natarajan L.V., Bunning T.J., White T.J. (2014). Color-Tunable Mirrors Based on Electrically Regulated Bandwidth Broadening in Polymer-Stabilized Cholesteric Liquid Crystals. ACS Photonics.

[78] Lu H.B., Wang Q., Zhu M.M., Huang P., Xu M., Qiu L.Z., Zhu J. (2022). Electrically controllable reflection bandwidth polymer-stabilized cholesteric liquid crystals with low operating voltage. Liq. Cryst..

[79] Lee K.M., Bunning T.J., White T.J., McConney M.E., Godman N.P. (2021). Effect of Ion Concentration on the Electro-Optic Response in Polymer-Stabilized Cholesteric Liquid Crystals. Crystals.

[80] Li Z.Z., Lan R.C., Bao J.Y., Hu W., Wang M., Zhang L.Y., Yang H. (2022). Tunable Circularly Polarized Luminescence with a High Dissymmetry Factor Emitted from Luminogen-Bonded and Electrically Controlled Polymer-Stabilized Cholesteric Liquid Crystals. ACS Appl. Mater. Interfaces.

[81] Zhang L., Nie Q., Jiang X.-F., Zhao W., Hu X., Shui L., Zhou G. (2021). Enhanced Bandwidth Broadening of Infrared Reflector Based on Polymer Stabilized Cholesteric Liquid Crystals with Poly(N-vinylcarbazole) Used as Alignment Layer. Polymers.

[82] Hu X., Zeng W., Zhang X., Wang K., Liao X., Jiang X., Jiang X.-F., Jin M., Shui L., Zhou G. (2020). Pitch Gradation by Ion-Dragging Effect in Polymer-Stabilized Cholesteric Liquid Crystal Reflector Device. Polymers.

[83] Hu W., Zhao H., Shan L., Song L., Cao H., Yang Z., Cheng Z., Yan C., Li S., Yang H. (2010). Magnetite nanoparticles/chiral nematic liquid crystal composites with magnetically addressable and magnetically erasable characteristics. Liq. Cryst..

[84] Kokkonen M., Talebi P., Zhou J., Asgari S., Soomro S.A., Elsehrawy F., Halme J., Ahmad S., Hagfeldt A., Hashmi S.G. (2021). Advanced research trends in dye-sensitized solar cells. J. Mater. Chem. A.

[85] Sharma K., Sharma V., Sharma S.S. (2018). Dye-Sensitized Solar Cells: Fundamentals and Current Status. Nanoscale Res. Lett..

[86] Liu Y.M., Yu L., Jiang Y., Xiong W.S., Wang Q., Sun J., Yang H., Zhao X.Z. (2016). Self-organized cholesteric liquid crystal polymer films with tunable photonic band gap as transparent and flexible back-reflectors for dye-sensitized solar cells. Nano Energy.

[87] Wu M., Shi Y., Li R., Wang P. (2018). Spectrally Selective Smart Window with High Near-Infrared Light Shielding and Controllable Visible Light Transmittance. ACS Appl. Mater. Interfaces.

[88] Loonen R., Singaravel S., Trcka M., Costola D., Hensen J.L.M. (2014). Simulation-based support for product development of innovative building envelope components. Autom. Constr..

[89] Khandelwal H., Schenning A.P., Debije M.G. (2017). Infrared regulating smart window based on organic materials. Adv. Energy Mater..

[90] Ranjkesh A., Choi Y., Huh J.W., Oh S.W., Yoon T.H.J.S.E.M., Cells S. (2021). Flexible, broadband, super-reflective infrared reflector based on cholesteric liquid crystal polymer. Sol. Energy Mater. Sol. Cells.

[91] Khandelwal H., Debije M.G., White T.J., Schenning A. (2016). Electrically tunable infrared reflector with adjustable bandwidth broadening up to 1100 nm. J. Mater. Chem. A.

[92] Hu X., Zeng W., Yang W., Xiao L., De Haan L.T., Zhao W., Li N., Shui L., Zhou G. (2019). Effective electrically tunable infrared reflectors based on polymer stabilised cholesteric liquid crystals. Liq. Cryst..

[93] Yang H., Mishima K., Matsuyama K., Hayashi K., Kikuchi H., Kajiyama T. (2003). Thermally bandwidth-controllable reflective polarizers from (polymer network/liquid crystal/chiral dopant) composites. Appl. Phys. Lett..

[94] Zhang W.X., Lub J., Schenning A., Zhou G.F., de Haan L.T. (2020). Polymer Stabilized Cholesteric Liquid Crystal Siloxane for Temperature-Responsive Photonic Coatings. Int. J. Mol. Sci..

[95] Rafayelyan M., Agez G., Brasselet E. (2017). Ultrabroadband gradient-pitch Bragg-Berry mirrors. Phys. Rev. A.

[96] Zhao Y.Z., Tian S.P., Wang Z.D., Zhu M., Ren H.P., Ma Q., Guo Z., Li K.X., Ding S.Y., Miao Z.C. (2017). A new-type composite film of cholesteric liquid crystal doped with PCBM for laser-damage prevention in both visible and near-infrared region. Mol. Cryst. Liq. Cryst..

